# Supplementary material for: Single-cell sortChIC identifies hierarchical chromatin dynamics during hematopoiesis
Source: Nat Genet. 2022 Dec 20;55(2):333–45. doi: 10.1038/s41588-022-01260-3 (PMC9925381; doi:10.1038/s41588-022-01260-3)
Supplement: Supplementary file 1 — Supplementary Figs. 1 and 2, Supplementary Tables 1–7, and Supplementary Note sections Discussion, Methods and Materials. [file 41588_2022_1260_MOESM1_ESM.pdf]

nature genetics

Technical Report

https://doi.org/10.1038/s41588-022-01260-3

Single-cell sortChIC identifies hierarchical chromatin dynamics during hematopoiesis

In the format provided by the authors and unedited

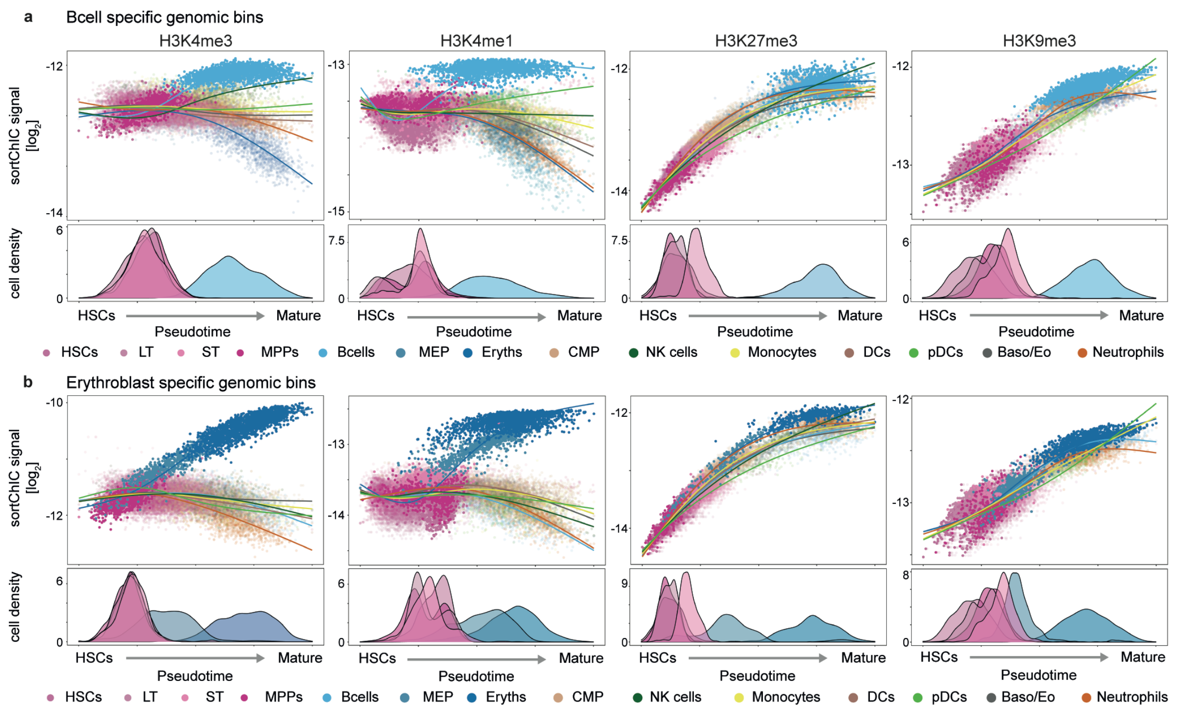

Supplementary Fig. 1: Pseudotime analysis from HSCs, committed progenitors, to differentiated cell types across dynamic 50 kb bins. (a) Mean sortChIC signal for bins that are upregulated in B cells relative to HSPCs across cell types for the four histone marks independently. Regions are defined for each histone modification separately (H3K4me3: 948 bins, H3K4me1: 4409 bins, H3K27me3: 2753 bins, H3K9me3: 3099 bins). Density plots below show the distribution of cell types along the B cell trajectory (HSCs, LTs, STs, MPPs, B cells). Cubic spline a fit across pseudotime for each trajectory. (b) Same as a but for bins upregulated in erythroblasts relative to HSCs. Density plots below (HSCs, LTs, STs, MPPs, MEP, Erythroblasts). Regions are defined for each histone modification separately (H3K4me3: 697 bins, H3K4me1: 3492 bins, H3K27me3: 2252 bins, H3K9me3: 3498 bins).

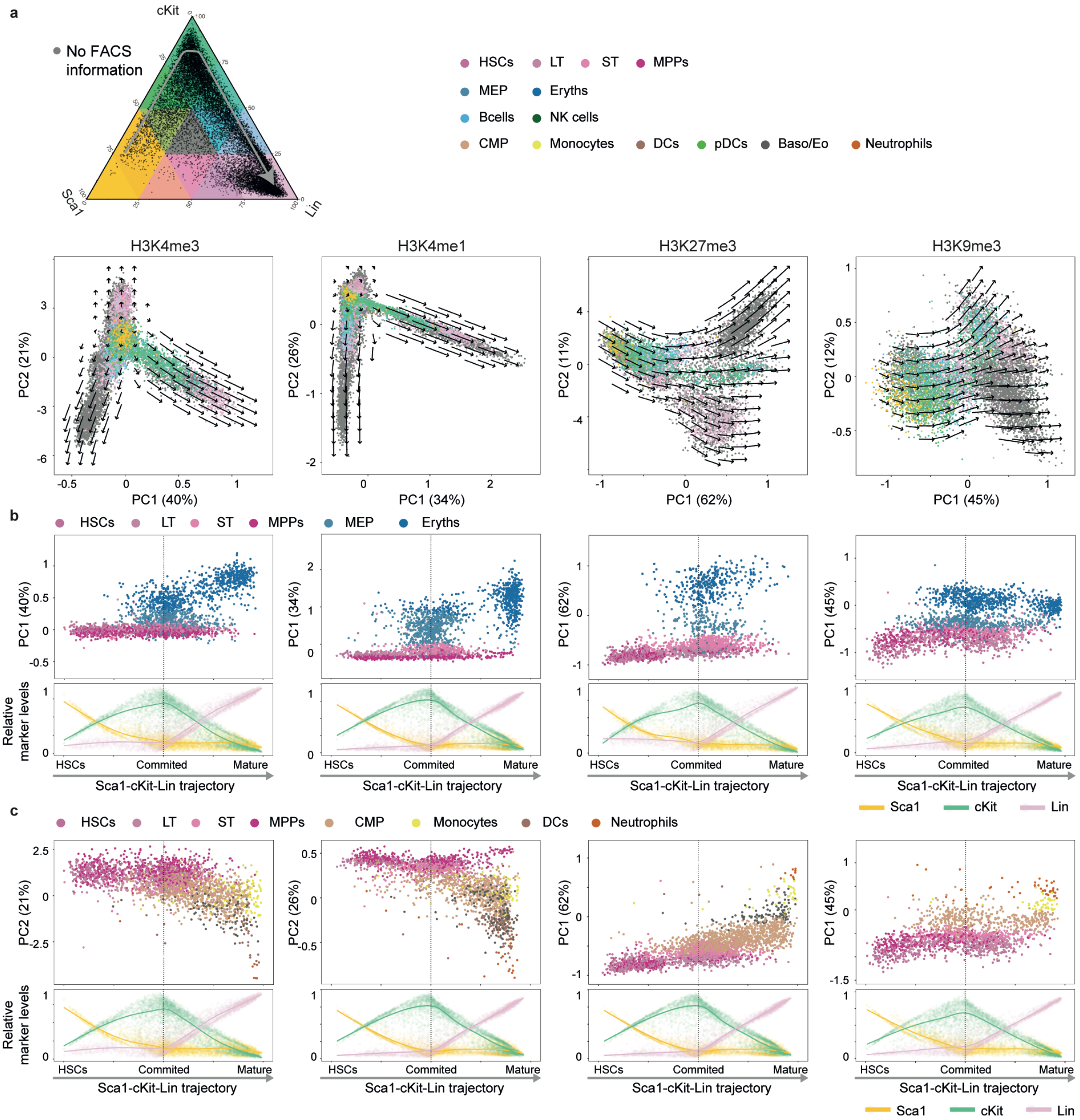

Supplementary Fig. 2: Global chromatin changes along the FACS-defined Sca1-cKit-Lin trajectory. (a) First two principal components for H3K4me3, H3K4me1, H3K27me3, and H3K9me3. Cells are colored by relative levels of Sca1, cKit, and Lin in. (b) Cells arranged along Sca1-cKit-Lin trajectory plotted against the main principal component showing variability along the erythroblast trajectory. (c) Same as (b) but filtering for cells along the neutrophil trajectory. For H3K4me1 and H3K4me3 the main principle component showing variability along the neutrophil trajectory is PC2.

Supplementary Discussion

Technologies to profile histone modifications in single cells by sequencing are still in their infancy, but has the potential to unlock the spectrum of chromatin states in the genome of individual cells. The ideal assay strives to have high sensitivity, high throughput, and robustness in both active and repressive chromatin states. Current techniques to map histone modifications in single cells use one of three approaches: ChIP-based, pA-Tn5-based, and pA-MNase-based. ChIP-based strategies utilize microfluidics systems or combinatorial barcoding to overcome the low sensitivity of ChIP [21–23]. pA-Tn5-based strategies profile histone modifications with very high throughput, but due to the intrinsic affinity of Tn5 to open chromatin regions [29–34], high specificity can so far only be achieved at the cost of some sensitivity. pA-MNase-based methods profile histone modifications with high sensitivity, and have robust detection of modifications associated with euchromatic regions as well as heterochromatic regions, but has generally less throughput compared with Tn5-based methods [26–28]. SortChIC is a unique single-cell method that combines cell enrichment to greatly enhance throughput of rare cells, while achieving high sensitivity and robustness to profile active and repressive chromatin states (Extended Data Fig. 2, Supplementary Table 2), thereby complementing newly available high throughput methods[32].

This comprehensive profiling of rare progenitors and their multiple cell fates enables new systematic analyses, such as quantifying chromatin dynamics that are cell fate-independent during differentiation. This analysis reveals that cell fate-independent changes during differentiation occur frequently for repressive chromatin, while such changes for active chromatin are rare. Our strategy combines rare progenitor cell enrichment with comprehensive differentiated cell type profiling to allow systematic analysis of chromatin dynamics during differentiation into multiple cell fates.

Supplementary Table 4: sorted plates per condition

			H3K4me1	H3K4me3	H3K27me3	H3K9me3	H3K4me1+H3K9me3
K562	fixed		3	3	3	3	0
BM	nuclei	unenriched	3	3	0	3	0
		lin-	3	3	0	3	0
		LSK	3	3	0	3	0
	fixed	SL0	19	17	18	17	0
		SL1	2	2	2	2	0
		SL2	2	2	2	2	0
		SL3	2	2	2	2	10
		SL4	5	5	5	5	5
		SL5	10	10	10	10	0
			49	47	39	47	15
		cells sorted	18424	17672	14664	17672	5640
		cells in UMAP	11933	12380	8128	8886	

Gray cells indicate experiments performed with monoclonal antibody for this mark

Supplementary Table 5: end repair mix

	Volumes per well (nl)
Klenow large (NEB, M0210L)	2.5
T4 PNK (NEB, M0201L)	2.5
dNTPs 10 mM (Promega, U1515)	6.0
ATP 100 mM (part of Thermo Fisher Scientific, R0441)	3.5
MgCl2 25 mM (part of Thermo Fisher Scientific, 4398828)	10.0
PEG8000 50% (Promega, V3011)	7.5
PNK buffer 10X (NEB, B0201S)	35.0
BSA 20 ng/ml (NEB, B9000S)	1.8
Nuclease-free water (Invitrogen, AM9932)	81.3
Total	150.0

Supplementary Table 6: A-tailing mix

	Volumes per well (nl)
AmpliTaq 360 (Thermo Fisher Scientific, 4398828)	1.0
dATPs 100mM (part of Promega, U1335)	1.0
KCl 1 M (Thermfisher, AM9640G)	25.0
PEG8000 50%	7.5
BSA 20 ng/ml	0.8
Nuclease-free water	114.8
Total	150.0

Supplementary Table 7: Adaptor ligation mix

	Volumes per well (nl)
T4 ligase (400K Units/ml, NEB, M0202L)	25.0
$MgCl_2$ 1 M (ThermoFisher, AM9530G)	3.5
Tris 1 M pH 7.5 (ThermoFisher, 15567027)	10.5
DTT 0.1M (Invitrogen, 15846582)	52.5
ATP 100 mM	3.5
PEG8000 50%	10.0
BSA 20 ng/ml	1.0
Nuclease-free water	44.0
Total	150.0

Supplementary Methods

Pa-MN production

JM101 bacteria were transformed with pK19pA-MN (Addgene plasmid # 86973). Bacteria are grown in LB media containing 50 mg/l kanamycin and protein production is activated at OD=0.4 by adding IPTG to 2mM final concentration and harvested after 2 h. For each 200 ml starting culture, bacteria are in centrifuged and resuspended in 10 ml TEN (10 mM Tris-HCl, pH 7.5, 1 mM EDTA, and 150 mM NaCl) containing 5 mM DTT. Bacteria were lysed by adding 1 mg chicken egg white lysozyme (Sigma) and incubation for 10 min at 4°C and sonication using a Branson SFX250. Debris was removed by centrifugation at 17000 rcf for 20 min at 4 °C. Protein was recovered from the supernatant using IgG Sepharose® 6 Fast Flow (Sigma Aldrich) following manufactures instructions, with the addition of 0.03% Empigen to the TEN based washing steps. After elution with 0.5ml 0.5 M HAc/ NH4Ac, pH 3.4 and neutralized by addition of 5M NaOH. After determining the protein concentration, Glycerol is added to a final concentration of 50% and aliquots are stored at -80 °C.

Data preprocessing

We developed a preprocessing pipeline called SingleCellMultiOmics (verision v.0.1.25) to process sortChIC data (https://github.com/BuysDB/SingleCellMultiOmics/wiki). The pipeline for sortChIC takes raw fastq files through the following software:

Demultiplexing: demux.py from SCMO v0.1.25

Adapter trimming: cutadapt (version 3.5)

Mapping: bwa (Version: 0.7.17-r1188)

Molecule assignment: bamtagmultiome.py (SCMO v0.1.25)

Count table generation: bamToCountTable.py (SCMO v0.1.25)

Latent Dirichlet allocation: from topicmodels version 0.2-12

Fastq files were demultiplexed by matching to an 8 nt cell barcode found in read 1 (R1). The 3 nt UMI was placed into the fastq header using demux.py, then the fastq files were trimmed using cutadapt with parameters indicated in 2-trim-fastq.sh in the example_processing_pipeline git repository. We mapped each read pair to a genomic location using bwa version 0.7.17-r1188 to the mm10 mouse genome Ensembl release 97. The cut site is defined as the genomic mapping location of the second base in R1. The ligation motif is defined as the two bases flanking the MNase cut site.

Assignment of read pairs to molecules is performed by pooling all read pairs that share the same UMI, cell barcode, and MNase cut site in a window of 1 kb.

We discarded read pairs if reads have:

- mapping quality scores (MAPQ) below 40,
- alternative hits at a non-alternative locus,
- mapped to separate locations beyond the expected insert size range,
- soft clips,
- more than 2 bases that differed from the reference,
- indels,
- mapping to a blacklist region (http://mitra.stanford.edu/kundaje/akundaje/release/blacklists/).

We selected cells with more than 500 total unique cuts for H3K4me1 and H3K4me3, and more than 1000 total unique cuts for H3K27me3 and H3K9me3. Cells also needed to have more than 50% of their cuts occur in an “AT” context. We also counted cut fragments that map in 50 kb nonoverlapping bins genome-wide, and calculated the fraction of bins that contains exactly zero cuts. Cells with a small fraction of zero cuts relative to other cells are more likely to have unspecific cuts. For each mark, we removed cells with a fraction of zero cuts that was below 2 standard deviations from the mean across all cells.

Comparison of sortChIC data with other single-cell chromatin profiling assays

In order to perform a fair comparison of sortChIC data with other similar assays, we downloaded the raw data from Bartosovic et. al. (GSE163532), Grosselin et. al. (GSE117309), Ku et. al. 2019 (GSE105012), Wu. et. al. (GSE139857), Kaya-Okur et. al. (GSE124557), and Ku et. al. 2021 (GSE139857), from GEO, and mapped and quantified them using the pipelines described by the authors in the original study. In short, we used cell-ranger atac (v2.0.0) for Bartosovic and Wu. et. al., scChIP-seq pipeline (https://github.com/vallotlab/scChIPseq) for Grosselin et. al., the iscChIC scripts (https://github.com/wailimku/iscChIC-seq) for Ku et. al. 2021, and the bowtie2 mapping command reported in Kaya-Okur and Ku et. al. 2019. Due to updated version of the deduplication in cell-ranger atac (v2.0.0) and the scChIP-seq pipeline (Grosselin et. al.), we obtained counts higher than originally reported. We used the de-duplicated BAM files for the comparison in Supplementary Fig. 1f-g. For the studies where instructions to obtain a PCR de-duplicated files were absent (Kaya-Okur et. al., Ku et al 2019, Ku et. al. 2021), we used UMI-tools (v1.0.0) for deduplication. We also used UMI-tools for the de-duplication of sortChIC data before counting. For other studies the duplicate marked/removed files from the original pipeline were used.

For the analysis of enrichment, we took the de-duplicated counts in 50kb bins, and subsetted for 1 sample per batch (Bartosovic: cell_line_mix_v1, brain_P15_GFP_pos; Grosselin: jurkat-ramos, HBXc-95, Wu21: GBM, sortChIC: BM-SL-5, K562-all). We then calculated the Fraction of reads in the top 25% bins (ranked using the mean signal of cells), and Gini scores per cell. If Xf_i is the count for bin i , the gini score for cell j is defined as:

$$gini(C_j) = \frac{\sum_{i=1}^{n} (2i - n - 1) * Xf_i, c_j}{n \sum_{i=1}^{n} Xf_i, c_j}$$

Dimensionality reduction based on multinomial models

We counted the number of cuts mapped to peaks across cells and applied the latent Dirichlet allocation (LDA) model [39], which is a matrix factorization method that models discrete counts

across predefined regions as a multinomial mixture model. LDA can be thought of as a discrete version of principal component analysis (PCA), replacing the normal likelihood with a multinomial one [76].

LDA models the genomic distribution of cuts from a single cell using a hierarchy of multinomials:

1. $\vec{V}_k \sim$ Dirichlet(δ) to specify the distribution over genomic regions for each topic *k* (length G genomic regions).
2. $\vec{U}_i \sim \mathrm{Dirichlet}(\alpha)$ to specify the distribution over topics for a cell *i* (length K topics).

To generate the genomic location of the *j*th read in cell *i*:

3. Choose a topic $z_{i,j} \sim$ Multinomial($\vec{U}_j, 1$)
4. Choose a genomic region $w_{i,j} \sim$Multinomial($\vec{V}_{z_{i,j}}, 1$)

We used the LDA model implemented by the *topicmodels* R package [77], to infer the cell-to-topic matrix (analogous to the scores matrix in PCA) and topic-to-region matrix (analogous to the loadings matrix in PCA) using Gibbs sampling with hyperparameters α =50/K, δ =0.1, where *K* is the number of topics. We used K=30 topics for all of our analyses.

Defining eight sets of blood cell type-specific genes for cell typing

We defined cell type-specific genes for cell type calling by counting reads at +/- 5 kb centered at annotated transcription start sites (RefSeq TSS, https://ccg.epfl.ch/mga/mm10/refseq/refseq.html). We applied LDA to the resulting count matrix for H3K4me1, H3K4me3, and H3K27me3 (we ignored H3K9me3 here because H3K9me3 marks mostly AT-rich, gene-poor regions). We found eight topics that defined the eight cell types in the data. For each topic, we took the top 150 TSS loadings to make eight sets of genes defining the cell types in the data. For basophils, we took the top 50 loadings and manually added *Il4*, *Il6*, *Cpa3,* and *Il1r1* because we found that this smaller set was more

specific to basophils. To compare this set of TSSs to publicly available scRNA-seq data (Baccin et al 2020) [4], we took each TSS and assigned it to the corresponding gene.

Batch correction in dimensionality reduction

Initial LDA of the count matrix revealed batch effects in H3K4me1 and H3K9me3 between cell types of plates that contained only one sorted type (i.e., entire plate was either unenriched, lineage-negative, or LSK cells, referred to as "single-type") and cell types from plates that contained a mixture of unenriched and non-mature cells (referred to as "balanced"). We corrected batch effects in H3K4me1, H3K4me3, and H3K9me3. Since H3K27me3 did not have single-type plates, we did not correct batch effects in H3K27me3. We considered balanced plates as the reference for differences between cell types, and corrected deviations in single-type plates to match the balanced plates. We used the imputed sortChIC-seq signal inferred from LDA as a denoised signal *Y* for each genomic region *g* for every cell *c*:

$$Y_{g,c} = \log_2\left(\sum_{k=1}^{K} V_{g,k} U_{k,c}\right)$$

We modeled the cell type-specific batch effect using a linear model for each genomic region. The model infers the effect of a cell *c* belonging to batch *b* and cell type *d*:

$$Y_c(b,d) = \beta_0 + \beta_1 \mathbf{1}_s(b) + \sum_{j=1}^{J} \beta_{2,j} \mathbf{1}_j(d) + \beta_{3,j}\ \left(\mathbf{1}_j(d) \cdot \mathbf{1}_s(b)\right) + \epsilon$$

Where:

$\mathbf{1}_s(b)$ is an indicator variable equal to 1 if the cell is from a single-type plate (batch *s*), otherwise 0.

$\mathbf{1}_j(d)$ is equal to 1 if cell belongs to cell type *j*, otherwise 0.

β_0 is the intercept of the model.

β_1 is the global effect (i.e., independent of cell type) from a cell being from batch s (single-type plate).

$\beta_{2,j}$ is the effect from a cell belonging to cell type j.

$\beta_{3,j}$ is the interaction effect from a cell belonging to cell type *j* and being from batch s (single-type plate).

ϵ is Gaussian noise.

We inferred the effects for each genomic region using $\mathrm{lm}()$ in R with the formula syntax:

$$Y \sim 1 + \text{batch} + \text{celltype} + \text{batch:celltype}$$

and estimated the batch-corrected signal:

$$\tilde{Y} = \begin{cases} Y & \text{if cell from complete plate} \\ Y - \beta_1 - \beta_{3,j} & \text{if cell from single-type plate} \end{cases}.$$

For cells that belong to complete plates, $\mathbf{1}_s(b) = 0$. Therefore, this batch-correction only corrects signal from cells belonging to single-type plates. In the bone marrow analysis this corresponds to nine plates for H3K4me1, H3K4me3, and H3K9me3.

The corrected signal is used to refine the cell-to-topic and topic-to-region matrix by GLM-PCA [40]. We applied SVD to the batch-corrected matrix to use as initializations for U and V, and included batch ID as cell-specific covariates (in glmpca R package: glmpca(), with fam=”poi”, minibatch=”stochastic”, optimizer=”avagrad”, niterations = 500). The batch-corrected U matrix is then visualized with uniform manifold approximation and projection (UMAP).

Differential histone mark levels analysis

To calculate the fold change in histone mark levels at a genomic region between a cell type versus HSPCs, we modeled the discrete counts *Y* across cells as a Poisson regression. We fitted a null model, which is independent of cell type, and a full model, which depends on the cell type and compared their deviances to predict whether a region was "changing" or "dynamic" across cell types. We implemented the model in R using glm().

We used the glm() implementation in R with the formula syntax for the full and null model:

$$\text{Full model: counts}{\sim}1 + \text{batch} + \text{celltype} + \text{offset}(\log(\text{totalcounts}))$$

$$\text{Null model: counts}{\sim}1 + \text{batch} + \text{offset}(\log(\text{totalcounts})).$$

We used *G* as a deviance test statistic:

$$G = D_{full} - D_{null},$$

where the deviance is two times the log-likelihood, which for Poisson is:

$$D = 2\sum_{i=1}^{n}\{Y_i \log(Y_i/\mu_i) - (Y_i - \mu_i)\}$$

For the full model, the logarithm of the expected value μ is:

$$\log(\mu) = \beta_0 + \beta_1 \mathbf{1}_s + \sum_{j=1}^{J} \beta_{2,j} \mathbf{1}_j,$$

While for the null model, it is:

$$\log(\mu) = \beta_0 + \beta_1 \mathbf{1}_s,$$

We fitted the model such that the estimated $\log_2$ fold change of a cell type j, $\frac{\hat{\beta}_{2,j}}{\log(2)}$, is always relative to HSPCs.

Under the null hypothesis, G is chi-squared distributed with degrees of freedom equal to the difference in the number of parameters in the two models. We use this test statistic to estimate a p-value and infer whether a 50kb bin is "changing" or "dynamic" across cell types. For H3K4me1, H3K4me3, and H3K27me3, we used a Benjamini-Hochberg adjusted p-value of $q<10^{-50}$. For H3K9me3, where fold changes were generally smaller, we used $q<10^{-9}$. This separate cutoff for H3K9me3 allowed comparable number of differential bins for downstream analysis.

Calculating bins that change independent of cell type

We defined "changing bins" or "dynamic bins" using the deviance test statistic, detailed above in "Differential histone mark analysis", using a q-value$<10^{-50}$ for H3K4me1, H3K4me3, and H3K27me3, and $q<10^{-9}$ for H3K9me3. We defined these bins to be changing in a cell fate-independent manner if the estimated cell type effect, $\hat{\beta}_{2,j}$, was either greater than 0 for all cell types (gained relative to HSPCs) or less than 0 for all cell types (lost relative to HSPCs).

Predicting Activities of Transcription Factors in Single Cells

We adapted MARA (Motif Activity Response Analysis) described in [42] to accommodate the sortChIC data. Briefly, we model the log imputed sortChIC-seq signal as a linear combination of TF binding sites and activities of TF motifs using a ridge regression framework:

$$\tilde{Y}_{g,c} = \sum_{m=1}^{M} N_{g,m} A_{m,c} + \epsilon$$

Where $\tilde{Y}_{g,c}$ is the batch-corrected sortChIC-seq signal in genomic region *g* in cell *c*; $N_{g,m}$ is the number of TF binding sites in region *g* for TF motif *m*; $A_{m,c}$ is the activity of TF motif *m* in cell *c*; ϵ is Gaussian noise. The L2 penalty for ridge regression was determined automatically using a 80/20 cross-validation scheme. Z-scores of motifs greater than 0.7 were kept as statistically significant motifs.

The single-cell motif activity, $A_{m,c}$, is then overlaid onto the UMAP to show cell type-specific activities.

For H3K4me1, we defined genomic regions based on peak calling from *hiddenDomains*. For repressive marks, where domains can be larger, we used 50 kb bins that were significantly changing across cell types as genomic regions.

Creating the TF binding site matrix

We predicted the TF binding site count occurrence under each peak using the mm10 Swiss Regulon database of 680 motifs (http://swissregulon.unibas.ch/sr/downloads). We used the Motevo method to predict transcription factor binding sites. Posterior probabilities < 0.1 are rounded down to zero.

Joint H3K4me1 and H3K9me3 analysis by double incubation

To simultaneously infer the H3K4me1 and H3K9me3 cluster from single-cell double-incubated cuts, we focused on regions that were most informative to distinguish between clusters in H3K4me1 and in H3K9me3. For H3K9me3, we used 6085 statistically significant changing bins ($q<10^{-9}$, Poisson regression). For H3K4me1, we used regions near cell type-specific genes that were used to determine cell types from the data (811 regions). Since H3K4me1 had strong signal at both the TSS and gene bodies, we defined regions for each gene from transcription start site (TSS) to either its end site or 50 kb downstream of the TSS, whichever is smaller. We counted cuts mapped to these 6896 regions for H3K4me1, H3K9me3, as well as H3K4me1+H3K9me3 cells.

For a single cell, we assumed that the vector of H3K4me1+H3K9me3 counts $\vec{y}$ was generated by drawing *N* reads from a mixture of two multinomials, one from a cell type *c* from H3K4me1 (parametrized by relative frequencies $\vec{p}_c$) and one from a lineage *l* from H3K9me3 (parametrized by relative frequencies $\vec{q}_l$):

$$\vec{y}|c, l, w \sim \text{Multinomial}(w\vec{p}_c + (1 - w)\vec{q}_l, N),$$

where *w* is the fraction of H3K4me1 that was mixed with H3K9me3.

Genomic region probabilities $\vec{p}_c$ and $\vec{q}_l$ were inferred by the single-incubated data by averaging the imputed signal across cell types:

$$q_{l,g} = \frac{1}{|D_l|} \sum_{d=1}^{|D_l|} \sum_{k=1}^{K} V_{g,k} U_{k,d}$$

where D_l is the set of cells that belong to lineage l. *V* and *U* are estimated from LDA.

The log-likelihood for the H3K4me1+H3K9me3 counts coming from cluster pair (*c, l*), can be defined as:

$$\text{LL}_{(c,l)} \propto \sum_{g=1}^{G} y_g \log\big(wp_{c,g}(1 - w)q_{l,g}\big),$$

where g is a genomic region.

To assign a cluster pair to a double-incubated single cell, we calculated the log-likelihood for each possible pair (we had four lineages from H3K9me3 and eight clusters from H3K4me1, creating a 32 possible pairs) and selected the pair with the highest log-likelihood. We used the Brent method implemented in R (*optim*) to infer *w* that maximizes the log-likelihood for each pair.

Imputing Sca1-cKit-Lin marker levels

SL3-sorted plates have Sca1 and cKit marker levels (n=1842 cells), SL4-sorted plates have Sca1, cKit, and Lin marker levels (n=5247 cells), and SL5-sorted plates have cKit and Lin marker levels (n=10558 cells). To impute the missing Lin levels in SL3 and missing Sca1 levels in SL5, we used the SL4-sorted plates as a reference. For each cell in SL3 or SL5, we found the top 10 nearest neighbors to SL4 cells in the chromatin space (Euclidean distance in the sortChIC signal) and inferred Lin or Sca1 levels as the mean marker levels of the top 10 nearest neighbors to SL4 cells. Centered and scaled Sca1, cKit, and Lin marker levels are transformed to values between the interval [0, 1] by the Softmax function.

Reference-based cell typing using multinomials

We use the FACS-gated HSCs, ST-HSCs, LT-HSCs, MPPs, MEPs, CMPs, cDCs, monocytes, neutrophils, erythroblasts, pDCs, NK cells, and B cells as reference cell types for annotation. We also add basophils identified from our initial unsupervised clustering analysis as a reference cell type. We assume the vector of counts $\vec{y}$ of total counts *N* generated from a cell *c* belonging to cell type *s* is multinomial distributed,

$$(\vec{y}_c \,|Z_c = s) \sim \mathrm{Multinomial}(N, \vec{p}_s)$$

Where $\vec{p}$ are event probabilities ($\sum p_i = 1$).

We use outputs from LDA to infer $p_g|Z = s$ for gene *g* and cell *s* by taking the mean probabilities across cells that belong to cell type *s, {S}*.

$$p_g|s = \frac{1}{|S|}\sum_{c\in\{S\}}\sum_{k=1}^{K} V_{g,k}U_{k,c}$$

Where *k=30,* the number of latent factors (topics) used in the LDA model. *V* (dimensions number of genes by number of latent factors) and *U* (dimensions number of latent factors by number of cells) are factorized matrices inferred by LDA. *{S}* are cells that belong to cell type *s* (for example, all cells FACS-sorted for CMPs would belong to a CMP set).

Once we have the $\vec{p}_s$ for each cell type s, we can calculate the log-likelihood of an unlabeled cell to belong to cell type *s* given a raw count vector $\vec{y}$,

$$\log \Pr(\vec{y}|Z = s) = LL_s \propto \sum_{g \in \{G\}} y_g \log p_{g,s} \,.$$

We can then calculate the probability of a cell *c* to belong to cell type *s:*

$$\Pr(Z_c = s|\vec{y}_c) = \frac{\Pr(\vec{y}_c|Z_c = s)\Pr(Z_i = s)}{\sum_{s' \in \{celltypes\}} \Pr(\vec{y}_c|Z_c = s')}.$$

We assign each cell to the most probable cell type label. Subroutines for this method is implemented as an R package: https://github.com/jakeyeung/annotatecelltypes

Inferring pseudotime across different differentiation trajectories

We manually selected two principal components (PCs) for each cell type trajectory, selecting components that show large variation from progenitors (HSCs, LT, ST, MPPs), committed progenitors (e.g., CMPs, MEPs), to mature cell types (e.g., neutrophils, DCs, basophils, monocytes, pDCs, NK cells, B cells) of interest. The selected cells are then fit along a one-dimensional vector on the 2D space by finding the main eigenvector along the trajectory. For all trajectories, we used HSCs, LTs, STs, and MPPs to define the root. For myeloid cell types (neutrophils, DCs, basophils, and monocytes), we also included CMPs as committed progenitors. For erythroblasts, we included MEPs as commited progenitors. This eigenvector defines the pseudotime, with the end closest to HSCs defined as the root (pseudotime = 0) and the end closest to the mature cell type defined as the end state (pseudotime = 1).

Chromatin velocity in each histone modifications

After assigning each cell to a trajectory (cells annotated as HSCs, LTs, STs, and MPPs are annotated to all trajectories, while each mature cell type is assigned to one trajectory), we take each dynamic bin and fit a trajectory-specific cubic spline across pseudotime using the mgcv R package with the formula:

mgcv::gam(formula = signal ~ s(pseudotime, k = 4, bs = "cs", by = trajectory), gamma = 10, method = "REML")

We calculate the derivatives of this cubic spline for each bin along pseudotime using finite differences, as implemented by the gratia R package.

For each cell and for each bin, we take the derivatives of the cubic spline function with respective to pseudotime and project to a future pseudotime step of 0.01. Cells that are assigned to multiple trajectories (e.g., HSCs are assigned to all trajectories) take the mean of the future sortChIC signal across different trajectories. The high-dimensional predicted sortChIC signal at the future pseudotime step is then projected onto the PCA. We use the velocity grid flow visualization as implemented in velocity[78] to visualize the velocity vectors on the PCA space.

Supplementary Materials

Histone mark Antibodies

H3K4me1, ab8895 (Abcam), Lot: GR3206285-1, 1:200

H3K4me3, 07-473 (Merck), Lot: 3093304, 1:200

H3K4me3, MA5-11199 (Thermo Fisher), clone: G.532.8, 1:400

H3K9me3, ab8898 (Abcam), Lot: GR3217826-1, 1:100

H3K9me3, MA5-33395 (Thermo Fisher), clone: RM389, 1:100

H3K27me3, 9733S (NEB), clone: C36B11, 1:100

Surface marker Antibodies

SL0

Streptavidin-PE (Biolegend, 405203, 1:5000)

C-kit-APC (Biolegend, 105811, clone: 2B8, 1:800)

Sca1-PeCy7 (Biolegend, 108113, clone: D7, 1:400)

SL1

NK1-Alexa488 (Biolegend, 108717, clone: PK136, 1:400)

Ter119-PE (Biolegend, 116207, clone: Ter-119, 1:2000)

CD19-Alexa647 (BD, 557684, clone: 1D3, 1:800)

CD3-APC-Cy7 (Biolegend, 100221, clone: 17A2, 1:100)

SL2

CD11b-APC-Cy7 (BD, 561039, clone: M1/70, 1:1600)

CD14-Alexa647 (BD, 565743, clone: rmC5-3, 1:800)

CD24-PE (Biolegend, 101807, clone: M1/69, 1:1000)

Gr1-Alexa488 (Thermofisher, 53-5931-80, clone: 53-5931-8 1:800)

SL3 (depletion of lin+ cells)

C-kit- BB700 (BD, 566414, clone: 2B8, 1:800)

Sca1-PeCy7 (Biolegend, 108113, clone: D7, 1:400)

Flt3-PE-Cy5 (ThermoFisher, 15-1351-82, clone: A2F10, 1:800)

CD150-PE (BD, 562651, clone: Q38-480, 1:400)

CD34- Alexa488 (ThermoFisher, 53-0341-82, clone: RAM34, 1:100)

SL4 (depletion of lin+ cells)

C-kit- BB700 (BD, 566414, clone: 2B8, 1:800)

Sca1-PE-Cy7 (Biolegend, 108113, clone: D7, 1:400)

FCgamma-APC (ThermoFisher, 17-0161-81, clone: 93, 1:800)

Streptavidin-PE Biolegend, 405203, 1:5000)

CD34- Alexa488 (ThermoFisher, 53-0341-82, clone: RAM34 1:100)

SL5

C-kit- BB700 (BD, 566414, clone: 2B8, 1:800)

Siglec- APC (ThermoFisher, 17-0333-80, clone: eBio440c, 1:200)

Streptavidin-PE Biolegend, 405203, 1:5000)

IL7 R- Alexa488 (ThermoFisher, 53-1271-82, clone: A7R34, 1:1600)

Primers

Adaptor design

Top strand

GGTGATGCCGGTAATACGACTCACTATAGGGAGTTCTACAGTCCGACGATCNNN*ACAC ACTA*T

Bottom strand

/5Phos/*TAGTGTGT*NNNGATCGTCGGACTGTAGAACTCCCTATAGTGAGTCGTATTACCG GC**GAGCTT**

Sequence features from left to right on the top strand:

Bases written in bold form a fork to prevent adaptor dimer- or multimerization. Bases in green represent T7 polymerase binding site for IVT based amplification. Bases in blue are the binding site (RA5) for the TruSeq Small RNA indexing primers (RPIx). The 3 random nucleotides underlined are the unique molecular identifier used for read deduplication and the 8 bases afterwards in italics represent the cell barcode which is different each of the 384 wells. For a full list of adaptors see Supplementary Table 3.

randomhexamerRT primer: GCCTTGGCACCCGAGAATTCCANNNNNN
