# Supplementary material for: Single-cell sortChIC identifies hierarchical chromatin dynamics during hematopoiesis
Source: Nat Genet. 2022 Dec 20;55(2):333–45. doi: 10.1038/s41588-022-01260-3 (PMC9925381; doi:10.1038/s41588-022-01260-3)
Supplement: Supplementary file 2 — Reporting Summary [file 41588_2022_1260_MOESM2_ESM.pdf]

nature portfolio

Corresponding author(s): Alexander van Oudenaarden

Last updated by author(s): Oct 25, 2022

Reporting Summary

Nature Portfolio wishes to improve the reproducibility of the work that we publish. This form provides structure for consistency and transparency in reporting. For further information on Nature Portfolio policies, see our Editorial Policies and the Editorial Policy Checklist.

Statistics

For all statistical analyses, confirm that the following items are present in the figure legend, table legend, main text, or Methods section.

n/a	Confirmed	
☐	☒	The exact sample size (*n*) for each experimental group/condition, given as a discrete number and unit of measurement
☐	☒	A statement on whether measurements were taken from distinct samples or whether the same sample was measured repeatedly
☐	☒	The statistical test(s) used AND whether they are one- or two-sided<br>*Only common tests should be described solely by name; describe more complex techniques in the Methods section.*
☒	☐	A description of all covariates tested
☐	☒	A description of any assumptions or corrections, such as tests of normality and adjustment for multiple comparisons
☐	☒	A full description of the statistical parameters including central tendency (e.g. means) or other basic estimates (e.g. regression coefficient) AND variation (e.g. standard deviation) or associated estimates of uncertainty (e.g. confidence intervals)
☐	☒	For null hypothesis testing, the test statistic (e.g. *F*, *t*, *r*) with confidence intervals, effect sizes, degrees of freedom and *P* value noted<br>*Give P values as exact values whenever suitable.*
☐	☒	For Bayesian analysis, information on the choice of priors and Markov chain Monte Carlo settings
☒	☐	For hierarchical and complex designs, identification of the appropriate level for tests and full reporting of outcomes
☐	☒	Estimates of effect sizes (e.g. Cohen's *d*, Pearson's *r*), indicating how they were calculated

Our web collection on statistics for biologists contains articles on many of the points above.

Software and code

Policy information about availability of computer code

Data collection	single cell FACS data was collected using BD FACS software (version 1.2.0.124)
Data analysis	Github repo of processing and analysis scripts: https://github.com/jakeyeung/sortChIC<br><br>bwa Heng et al., 2010 Bioinformatics Version: 0.7.17-r1188<br>topicmodels Hornik & Grun 2011 Journal of Statatistical Software Version: 0.2-12<br>Glmpca Townes et al. 2019 Genome Biology Version: 0.2.0<br>motevo Arnold et al. 2012 Bioinformatics Version: 1.11<br>hiddenDomains Starmer and Magnuson 2016 BMC Bioinformatics Version: 3.1<br>SingleCellMultiOmics https://github.com/BuysDB/SingleCellMultiOmics/wiki

For manuscripts utilizing custom algorithms or software that are central to the research but not yet described in published literature, software must be made available to editors and reviewers. We strongly encourage code deposition in a community repository (e.g. GitHub). See the Nature Portfolio guidelines for submitting code & software for further information.

Data

Policy information about availability of data

All manuscripts must include a data availability statement. This statement should provide the following information, where applicable:

- Accession codes, unique identifiers, or web links for publicly available datasets
- A description of any restrictions on data availability
- For clinical datasets or third party data, please ensure that the statement adheres to our policy

All data produced in this study are deposited onto GEO under accession GSE164779. Token “srmnyauiphmflmx”
Data for K562 specificity comparison:
H3K4me1, Peggy Farnham, ENCSR000EWC, pAb-037-050 (Diagenode), H3K4me3, Peggy Farnheim, ENCSR000EWA, 9751S (Cell Signaling)
H3K9me3, Bradley Bernstein, ENCSR000APE, ab8898 (Abcam), H3K27me3, Peggy Farnheim, ENCSR000EWB, 9733S (Cell Signaling)
Data for comparison with similar assays was downloaded from GEO: Bartosovic et. al. (GSE163532), Grosselin et. al. (GSE117309), Ku et. al. 2019 (GSE105012), Wu. et. al. (GSE139857), Kaya-Okur et. al. (GSE124557), and Ku et. al. 2021 (GSE139857)
Transcription factor binding motives were used from the mm10 Swiss Regulon database of 680 motifs (http://swissregulon.unibas.ch/sr/downloads)
bone marrow scRNAseq data was used from Giladi et al 2018 (GSE113495)

Field-specific reporting

Please select the one below that is the best fit for your research. If you are not sure, read the appropriate sections before making your selection.

☒ Life sciences ☐ Behavioural & social sciences ☐ Ecological, evolutionary & environmental sciences

For a reference copy of the document with all sections, see nature.com/documents/nr-reporting-summary-flat.pdf

Life sciences study design

All studies must disclose on these points even when the disclosure is negative.

Sample size	Sample sizes (number of single cells) were chosen based on number of cells per cluster and occurrence of new clusters. They were determined to be sufficient when each cell type specific cluster contained at least 20 cells, and no new cell clusters were detected after doubling sample size.
Data exclusions	Single cells were selected based on minimal unique fragments per cell (500 for H3K4me1 and H3K4me3, 1000 for H3K9me3 and H3K27me3), at least 50% of the reads per cell had to start with the MN specific AT bias and intra-chromsomal variance of maximally 2 fold above average. More details can be found in Supplementary fig1 and the methods section.
Replication	Results were reproducible over 2 independent biological replica (2 mice) and hundreds of linked biological replicas (thousands of cells)
Randomization	No randomization was performed as there were no treatments/experiments were performed on the animals before cell isolation
Blinding	No blinding was performed as only one animal strain was used and results from all experiments were analysed with the identical computational pipeline, ensuring no influence of the experimentalist on observed differences.

Reporting for specific materials, systems and methods

We require information from authors about some types of materials, experimental systems and methods used in many studies. Here, indicate whether each material, system or method listed is relevant to your study. If you are not sure if a list item applies to your research, read the appropriate section before selecting a response.

Materials & experimental systems

n/a	Involved in the study
☐	☒ Antibodies
☐	☒ Eukaryotic cell lines
☒	☐ Palaeontology and archaeology
☐	☒ Animals and other organisms
☒	☐ Human research participants
☒	☐ Clinical data
☒	☐ Dual use research of concern

Methods

n/a	Involved in the study
☐	☒ ChIP-seq
☐	☒ Flow cytometry
☒	☐ MRI-based neuroimaging

Antibodies

Antibodies used	For ChIP (sortChIC):

Antibodies used	H3K4mel, ab8895 (Abeam), Lot: GR3206285-1;<br>H3K4me3, 07-473 (Merck), Lot: 3093304;<br>H3K4me3, MA5-11199 (Thermo Fisher), clone: G.532.8, monoclonal;<br>H3K9me3, ab8898 (Abeam), Lot: GR3217826-1;<br>H3K9me3, MA5-33395 (Thermo Fisher), clone: RM389, monoclonal;<br>H3K27me3, 9733S (NEB), clone: C36B11, monoclonal<br>For Flow cytometry:<br>C-kit-APC, 105811 (Biolegend), clone: 2B8, monoclonal;<br>Scal-PeCy7, 108113 (Biolegend), clone: D7, monoclonal;<br>NK1-Alexa488, 108717 (Biolegend), clone: PK136, monoclonal;<br>Ter119-PE, 116207 (Biolegend), clone: Ter-119, monoclonal;<br>CD19-Alexa647, 557684 (BD), clone: 1D3, monoclonal;<br>CD3-APC-Cy7, 100221 (Biolegend), clone: 17A2, monoclonal;<br>CD11b-APC-Cy7, 561039 (BD), clone: M1/70, monoclonal;<br>CD14-Alexa647, 565743 (BD), clone: rmC5-3, monoclonal;<br>CD24-PE, 101807 (Biolegend), clone: M1/69, monoclonal;<br>Grl-Alexa488, 53-5931-80 (Thermo Fisher), clone: 53-5931-8, monoclonal;<br>C-kit-BB700, 566414 (BD), clone: 2B8, monoclonal;<br>Flt3-PE-Cy5, 15-1351-82 (Thermo Fisher), clone: A2F10, monoclonal;<br>CD150-PE, 562651 (BD), clone: Q38-480, monoclonal;<br>CD34-Alexa488, 53-0341-82 (Thermo Fisher), clone: RAM34, monoclonal;<br>FCgammaR-APC, 17-0161-81 (Thermo Fisher), clone: 93, monoclonal;<br>Siglec-APC, 17-0333-80 (Thermo Fisher), clone: eBio440c, monoclonal;<br>IL7R-Alexa488, 53-1271-82 (Thermo Fisher), clone: A7R34, monoclonal;
Validation	For the ChIP experiments<br>ab8895, 07-473, ab8898 and C36B11 where validated by provider by ChIP-seq in mouse<br>MA5-11199 and MA5-33395 where validated by provider for specificity by peptide array and Eliza respectively<br>all antibodies where further validated in this study by correlation to public ChIP data<br>data for ab8895, MA5-11199, MA5-33395 and 9733S are shown in extended Fig 1<br>All antibodies used for FACS analysis where validated by provider for FACS application in mouse

Eukaryotic cell lines

Policy information about cell lines

Cell line source(s)	K562, CCL-243 (ATCC)
Authentication	authentication by the supplier by Karyotyping and antigen expression
Mycoplasma contamination	Cell lines were tested every 2 months and never tested positive
Commonly misidentified lines<br>(See ICLAC register)	no commonly misidentified cell lines were used in this study

Animals and other organisms

Policy information about studies involving animals; ARRIVE guidelines recommended for reporting animal research

Laboratory animals	C57BL/6 mice, male, 3-months-old<br>All mouse studies were conducted in accordance with protocols approved by the ethics committee of the Hubrecht Institute in Utrecht. Mice were housed in a normal condition with 12:12h light: dark cycle in a temperature-controlled room with food and water ad libitum.
Wild animals	No wild animals were used in this study
Field-collected samples	no Field collected samples were used in this study
Ethics oversight	Dier Experimenten Commissie of the Royal Netherlands Academy of Arts and Sciences

Note that full information on the approval of the study protocol must also be provided in the manuscript.

ChIP-seq

Data deposition

☒ Confirm that both raw and final processed data have been deposited in a public database such as GEO.

☐ Confirm that you have deposited or provided access to graph files (e.g. BED files) for the called peaks.

Data access links<br>*May remain private before publication.*	https://www.ncbi.nlm.nih.gov/geo/query/acc.cgi?acc=GSE164779

Files in database submission	tagged bam files split by cell-type, batch corrected metadata and 50kb count tables are provided for each experiment, including K562 data for H3K4me1, H3K4me3, H3K9me3 and H3K27me3, as well as BM data merged from 2 independent biological replicas including H3K4me1, H3K4me3, H3K9me3, H3K27me3 and H3K4me1+H3K9me3 double incubation
Genome browser session (e.g. UCSC)	NA

Methodology

Replicates	Cell line experiments were performed in 3 independent biological replica (N=3) with hundreds of single cell observation (n=276) per replica. Mouse experiments were performed as 2 independent biological replica (N=2) with thousands of single cell observations (n=~3000) per replica.
Sequencing depth	Samples were sequenced to a depth of at least 1.5 oversequencing rate. Unique number of fragments per cell are provided in the manuscript. Sequencing was performed paired end 75 bp
Antibodies	H3K4me1, ab8895 (Abcam), Lot: GR3206285-1; H3K4me3, 07-473 (Merck), Lot: 3093304; H3K4me3, MA5-11199 (Thermo Fisher), monoclonal; H3K9me3, ab8898 (Abcam), Lot: GR3217826-1; H3K9me3, MA5-33395 (Thermo Fisher), monoclonal; H3K27me3, 9733S (NEB), monoclonal
Peak calling parameters	details on read mapping can be found in the methods section of the manuscript and https://github.com/BuysDB/SingleCellMultiOmics/wiki. For peak calling hiddenDomains was used with minimum peak length of 1000 bp
Data quality	NA
Software	bwa Heng et al., 2010 Bioinformatics Version: 0.7.17-r1188<br>topicmodels Hornik & Grun 2011 Journal of Statatistical Software Version: 0.2-12<br>Glmpca Townes et al. 2019 Genome Biology Version: 0.2.0<br>motevo Arnold et al. 2012 Bioinformatics Version: 1.11<br>hiddenDomains Starmer and Magnuson 2016 BMC Bioinformatics Version: 3.1<br>SingleCellMultiOmics https://github.com/BuysDB/SingleCellMultiOmics/wiki

Flow Cytometry

Plots

Confirm that:

☒ The axis labels state the marker and fluorochrome used (e.g. CD4-FITC).

☒ The axis scales are clearly visible. Include numbers along axes only for bottom left plot of group (a 'group' is an analysis of identical markers).

☒ All plots are contour plots with outliers or pseudocolor plots.

☒ A numerical value for number of cells or percentage (with statistics) is provided.

Methodology

Sample preparation	Where indicated mouse bone marrow cells were stained live with antibody combinations (see methods section) for surface marker staining. Cell culture cells and mouse bone marrow cells were further washed in PBS and fixed in 70% ethanol 1h at -20C. Cells were incubated over night with histone mark specific antibodies at 4C. Before the sorting unbound antibody was washed away and the Pa-MN fusion protein targeting and Hoechst staining was performed for 1h at 4C. After 2 extra washes cell were sorted in 384 well plates. More details see methods section.
Instrument	BD Influx™ Cell Sorter
Software	For FACS, BD FACS software (version 1.2.0.124) was used.
Cell population abundance	purity in post-sorted samples is determined by single cell sequencing and shown in the manuscript
Gating strategy	Gating strategy is illustrated in the manuscript Extended Figure 1a, 2a and 8a. For Cell lines are gated for SSC, FSC and a G1 hoechst stain. For Bone marrow cells the gating strategy includes FSC, SSC and a G1 hoechst staining for all sorted cells, +linage marker negative for lin- cells, +cKit positive and sca positive for LSK cells.

☒ Tick this box to confirm that a figure exemplifying the gating strategy is provided in the Supplementary Information.
